# The impact of COVID-19 lockdown restrictions on the short-term association between in-vehicle particulate pollutants and the respiratory health of Parisian taxi drivers

**DOI:** 10.5271/sjweh.4089

**Published:** 2023-09-01

**Authors:** Melissa Hachem, Lynda Bensefa-Colas, Isabelle Momas

**Affiliations:** 1 Paris University, CRESS - INSERM UMR_1153, INRAE, HERA team, Paris, France.; 2 Hôtel-Dieu Hospital, APHP. Centre – Paris University, Department of Occupational and Environmental Diseases, Paris, France.; # Equal contribution

**Keywords:** black carbon, lung function, mucosal irritation, professional driver, SARS-CoV-2, ultrafine particle

## Abstract

**Objective:**

This study assessed the short-term associations between in-vehicle ultrafine particles (UFP) and black carbon (BC) concentrations and irritation symptoms and lung parameters of taxi drivers, pre- and post-lockdown.

**Methods:**

As part of PUF-TAXI project, 33 taxi drivers were followed up during two typical working days. In-vehicle UFP and BC were continuously measured by monitoring instruments. Irritation symptoms during the working day were reported via an auto-questionnaire and lung function was assessed by a portable spirometer, pre- and post- work shift. Generalized estimating equations, adjusted for potential confounders, were used to study the association between air pollutants and health outcomes. Effect modification by measurement period (pre- and post-lockdown) was investigated.

**Results:**

UFP and BC concentrations inside taxi vehicles decreased significantly post- compared to pre-lockdown. Incidence of nose irritation was positively associated with in-vehicle UFP and BC levels pre-lockdown, when pollutant levels were higher, whereas no significant association was found post-lockdown. The decrease in the FEF_25–75%_ (forced expiratory flow at 25–75% of the forced vital capacity) during the working day was significantly associated with in-taxi UFP levels before but not after lockdown. No association was found with BC. By contrast, incidence of eye irritation was significantly inversely associated with in-vehicle humidity, regardless of pollutant concentrations and the measurement period.

**Conclusions:**

Our findings indicate that an upgrade in in-vehicle air quality could improve respiratory health. This study showed that the magnitude of the incidence of nasal irritation and decrease in lung function depends on UFP concentrations the commuters are exposed to.

During the current coronavirus disease (COVID-19) pandemic (1), national lockdown was imposed by many governments to prevent the spread of the virus. This intervention led to a substantial decrease in human activities and thus in anthropogenic emissions (2, 3). As a result, a remarkable decrease in outdoor air pollutant concentrations [nitrogen dioxide (NO_2_), carbon monoxide (CO), sulfur dioxide (SO_2_), particulate matter (PM_2.5_, PM_10_)] was observed (4–7). Hachem et al (8) also reported a significant (31–32%) reduction in ultrafine particle (UFP, <100 nanometers) and black carbon (BC) concentrations inside taxi vehicles after the first lockdown in the Paris area. This was mainly due to the decrease in traffic flow following the restrictions implemented during COVID-19 lockdown (8).

Short-term health effects of exposure to fine particulate pollutants (PM_2.5_ and PM_10_) have been extensively documented (9–11). However, less is known about the adverse health effects of UFP and BC (12). Hachem et al (13) found that an increase in interquartile range (IQR) of in-taxi UFP (20×10^3^ particles/cm^3^) and BC (1.75 µg/m^3^) was significantly associated with an increase in incident nasal irritation. Moreover, in-taxi UFP was associated with a decrease in the forced expiratory volume in one second (FEV_1_, the volume of air exhaled in the first second under force after a maximal inhalation), in the forced vital capacity (FVC, the total volume of air that can be exhaled during a maximal forced expiration effort) and in the forced expiratory flow at 25–75% of the FVC (FEF_25–75%_) (13, 14). Lammers et al (15) reported that a short-term exposure (5 hours) to UFP near a major airport in Amsterdam was associated with a decrease in FVC among healthy subjects (15). Furthermore, a significant association between 24-hour exposure to BC and a decrease in FVC was observed among schoolchildren with persistent respiratory symptoms. However, no such association was found with 24-hour UFP exposure (16). Although recent studies suggested short-term respiratory health effects of UFP and BC, evidence is still inconclusive due to the heterogeneity across studies regarding methods: study design and exposure/health assessment (17–19).

To address this gap in knowledge, we took advantage of the implementation of the first lockdown in the Paris area (from 17 March to 11 May 2020) to investigate whether the association between air pollutant concentrations and respiratory effects changed pre- and post-lockdown. In this respect, the PUF-TAXI project (Particules Ultrafines - TAXI), a repeated measurement study (2019–2020) aiming to (i) measure in-taxi traffic-related air pollutants (TRAP) (20) and (ii) evaluate the impact of this in-vehicle TRAP exposure on Parisian taxi drivers’ respiratory health (13), has provided a unique opportunity to address this issue. Hence, the aim of this study was to evaluate the short-term associations between in-vehicle UFP and BC concentrations and irritation symptoms and lung function, in the pre- and post-lockdown periods.

## Methods

### Study design and population

The present study was carried out among 33 Parisian taxi drivers who participated in the PUF-TAXI study during two working days: one before the lockdown (from 14vFebruary to 12 December in 2019; excluding July and August) and one after the COVID-19 pandemic lockdown (from 2 July to 9 December 2020) (supplementary material, www.sjweh.fi/article/4089, figure S1 and table S1). Some restrictions remained after lockdown such as teleworking and curfew to 21:00 hours. The inclusion and exclusion criteria were described elsewhere (13). On the sampling days, UFP and BC were measured inside taxi vehicles with no restrictions given to the drivers (ventilation settings, vehicle speed, areas covered by trips, opening/closing windows). Alongside the exposure assessment, taxi drivers reported the occurrence of irritation symptoms before and during the working days and respiratory lung parameters were measured pre- and post-work shifts. Each subject acted as his own control.

### Health assessment

An occupational physician conducted a standardized physical examination of taxi drivers at Hôtel-Dieu Hospital (Paris, France) to evaluate their general health. The taxi drivers’ weight and height were noted, and a skin prick test (SPT) was performed. The participants also filled out a questionnaire on their sociodemographic characteristics (age, sex); smoking status (smokers/ex-smokers/never smokers); comorbidities (respiratory and allergic diseases, hypertension, diabetes, etc.); and their work characteristics (job tenure, working hours per day, etc.).

After the medical consultation, the drivers participated in the field study on two working days. On the sampling days, taxi drivers underwent a spirometry test according to the European Respiratory Society and the American Thoracic Society guidelines (21), pre- and post- the work shift. The following lung function parameters were measured: FEV_1_, FVC and FEF_25–75%_. Participants also reported the occurrence and the severity of eye and nose irritations before and during the working days. Details regarding the health assessment are presented in Hachem et al (13).

### Exposure assessment

On sampling days, each taxi driver was equipped with a Diffusion Size Classifier Miniature (DiSCmini®; Wohlen, Switzerland, and commercialized by Testo SE & Co. KGaA, Titisee-Neustadt, Germany) and a microAeth® Model AE51 (AethLabs, San Francisco, California, USA) – linked to a GPS – to continuously monitor in-vehicle UFP and BC concentrations respectively on a 1-minute timebase. Temperature, humidity, and CO_2_ levels were measured by CP11® (Michel Instruments, Lyon, France). The measurement devices were placed inside a carry case on the shelf under the rear window of the vehicles (supplementary figure S2).

The taxi drivers self-reported characteristics of each trip (duration, the time when windows were open, the activation of air conditioning and air recirculation, and smoking inside the vehicle). Furthermore, the Atmo index from Airparif website (www.airparif.asso.fr) was used to estimate the global ambient air quality. The Atmo index – from 1 (very good) to 10 (very bad) – is based on four air pollutant levels: SO_2_, NO_2_, ozone (O_3_) and PM_10_. For each of these pollutants, a sub-index is calculated, and the daily Atmo index is equal to the highest. Data from the Paris Data Platform (opendata.paris.fr) was used to calculate average traffic flow for 24 hours during each working day.

Details of the exposure assessment protocol and the instrument calibration have been published previously (20) and are presented in the supplementary material.

### Statistical analysis

In-vehicle pollutant concentrations, measurement campaign characteristics, irritation symptoms (nose, eye), and lung function parameters (FEV_1_, FVC, FEF_25–75%_) of the taxi drivers pre- and post-lockdown were compared using the paired sample T-tests/Wilcoxon tests or McNemar’s paired sample test according to the type of variables and their distributions.

Since measurements were repeated (pre- and post-lockdown), the associations of in-vehicle UFP and BC exposure with (i) the incidence of irritation symptoms and (ii) the changes in lung function parameters (FEV_1_, FVC, FEF_25–75%_), during the working day, were assessed using generalized estimating equations (GEE) logistic and linear regressions, respectively. The dependent variables for each measurement period (pre- and post-lockdown) were defined as follows (i): an “incident irritation” is having nasal/eye problems during the working day or becoming worse compared to the start of the day (the symptom intensity scale during the working day > the symptom intensity scale at the start of the working day); and (ii) a “change in a lung function parameter” is the percent difference in the parameter at the end of the measurement day compared to the beginning of the day. All models were adjusted for relevant variables: age (years), body mass index (kg/m^2^), respiratory/allergic diseases (asthma or eczema or allergic rhinitis or a positive skin prick test), ambient temperature (°C), outdoor air quality (Atmo index), trip duration, in-taxi temperature (%) and/or humidity (%), time of air conditioning activation relative to trip duration (%). The selection of the covariates was based on the literature, the directed acyclic graph (DAG) built using DAGitty version 3.0 (22) and on the bivariate analysis.

Results were expressed as adjusted odds ratios (OR_adj_) for the logistic regressions and as adjusted beta coefficient (β_adj_) for the linear regression models with their 95% confidence intervals (CI).

Using an alpha of 0.15, multiplicative interactions were tested to explore potential modification effect by the period measurement (pre- and post-lockdown). When interactions were significant, we conducted a stratified analysis.

## Results

Supplementary table S2 shows the baseline characteristics of the participants.

### Change in working conditions

After lockdown, while some restrictions remained such as teleworking and a curfew of 21:00 hours, the working conditions of taxi drivers changed. A significant decrease in traffic flow occurred (P<0.0001, Wilcoxon test for paired sample) and taxi drivers made shorter trips both in time (P=0.001, paired T-test) and distance (P=0.004, Wilcoxon test for paired sample). They opened their cab windows more frequently to meet the French Ministry of Employment, Labor, and Social Inclusion's recommendations (P=0.039) (supplementary table S3).

### Change in UFP and BC concentrations inside taxis

**Figure 1:**
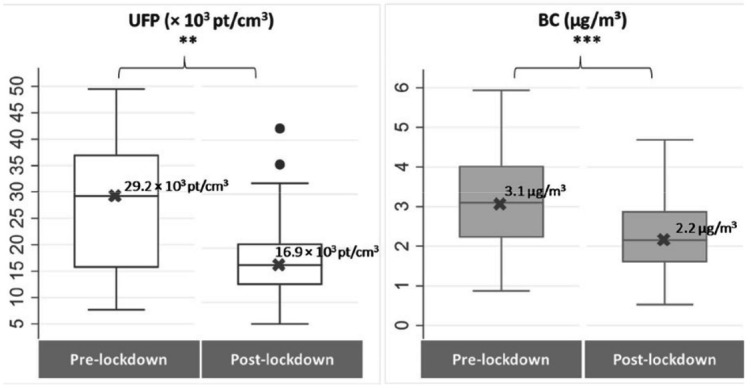
Distribution of ultrafine particles (UFP) and black carbon (BC) inside Parisian taxi vehicles pre- and post-lockdown, in the PUF-TAXI study. In each box, the thick middle line with the X symbol, top and bottom represent the median value, upper and lower quartile (75^th^ and 25^th^ percentile), respectively. In each box, the thick middle line with the X symbol, top and bottom represent the median value, upper and lower quartile (75^th^ and 25^th^ percentile), respectively. [BC= black carbon ; UFP=ultrafine particles]. ** P ≤ 0.01 (Wilcoxon test for paired sample); *** P ≤ 0.001 (paired T-test).

In-taxi UFP concentrations decreased significantly post-lockdown (median: 29.2×10^3^ versus 16.9×10^3^ particles/cm^3^) as well as in-taxi BC levels (median: 3.1 versus 2.2 µg/m^3^) (figure 1).

### Change in the associations between in-taxi UFP and BC concentrations and the incidence of irritation pre- and post-lockdown

**Figure 2:**
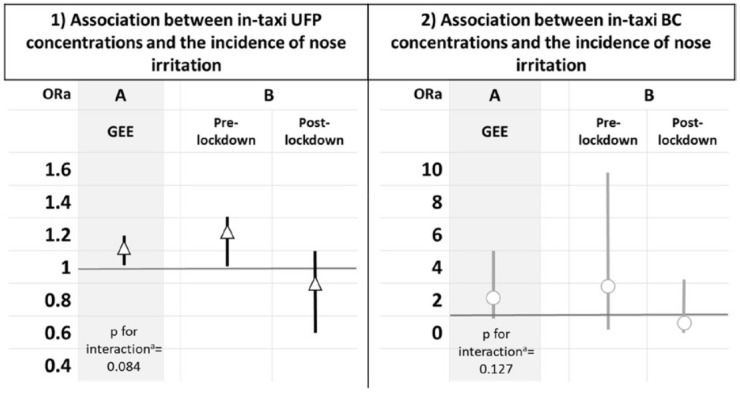
Associations between in-taxi (1) UFP (x103 particles/cm^3^) and (2) BC (µg/m^3^) concentrations and the incidence of nose irritation, in the PUF-TAXI study. [BC=black carbon; GEE=generalized estimating equations; UFP=ultrafine particles]. The odd ratios adjusted in models 1 & 2 were calculated for 1000 particles/cm^3^ increase in UFP and 1 μg/m^3^ increase in BC, respectively. Models in A were adjusted for the measurement period (before versus after the 1^st^ lockdown), ambient temperature (°C), outdoor air quality (Atmo index), trip duration, in-taxis temperature (%), time of air conditioning activation relative to the trip duration (%). Models in B were adjusted for the same variables as A except for the stratification variable (pre- versus post-lockdown). An incident nose irritation is defined as having nasal problems (sneezing, stuffy or runny nose, itchy nose) during the working day or getting worse compared to the start of the day (the symptom intensity scale during the working day > the symptom intensity scale at the start of the working day). a Interaction by the measurement period (pre- versus post-lockdown).

Overall, the incidence of nose irritation was significantly associated with in-taxi UFP concentration (OR_ad_ 1.13 (95% CI 1.01‒1.25); P=0.03) and tended to be related to in-taxi BC concentration (OR_adj_ 2.13 (95% CI 0.89‒ 5.04); P=0.08). However, these associations appeared to be modified by the measurement period (interaction P≤0.15) (figure 2). The incidence of nose irritation was positively associated with UFP concentration inside taxi vehicles before lockdown [OR_adj_=1.17 (95% CI 1.01‒1.35); P=0.03], whereas no association was found after lockdown [OR_adj_=0.86 (95% CI 0.63‒1.19); P=0.380]. The incidence of nose irritation and in-taxi BC concentration followed the same trend but did not reach the statistical significance (figure 2).

There was no association between the incidence of eye irritation and in-taxi particle concentrations. Nevertheless, the incidence of eye irritation appeared to be negatively associated with in-taxi humidity regardless of the measurement period (pre- or post-lockdown) (table 1).

**Table 1:** Associations between in-taxi UFP (×103 pt/cm^3^) and BC (μg/m^3^) concentrations and the incidence of eye irritation in the PUF-TAXI project. [BC=black carbon; CI=confidence interval; OR_adj_=adjusted odds ratio; UFP=ultrafine particles]

Eye irritation ^a^		Generalized estimating equations
		OR_adj_^b^ (95% CI)
Model 1	UFP (^×^103 pt/cm^3^)	1.08 (0.96‒1.20)
	In-taxi relative humidity (%)	0.73 (0.57‒0.93) ^c^
Model 2	BC (μg/m^3^)	1.51 (0.43‒5.30)
	In-taxi relative humidity (%)	0.79 (0.65‒0.95) ^c^

^a^ Eye problems (redness, watery or itchy eyes) during the working day or getting worse as the day progresses (the symptom intensity scale during the working day > the symptom intensity scale at the start of the working day). ^b^ Odd ratios in models 1 & 2 were calculated for 1000 particles/cm^3^ increase of UFP and 1 μg/m^3^ increase of BC, respectively. Models in A were adjusted for the measurement period (pre- vs post-lockdown), body mass index (kg/m^2^), outdoor air quality (Atmo index), trip duration, temperature inside taxi vehicles (°C). ^c^ P≤0.05.

Changes in the associations between in-taxi UFP and BC concentrations and the relative change in lung function parameters

**Figure 3:**
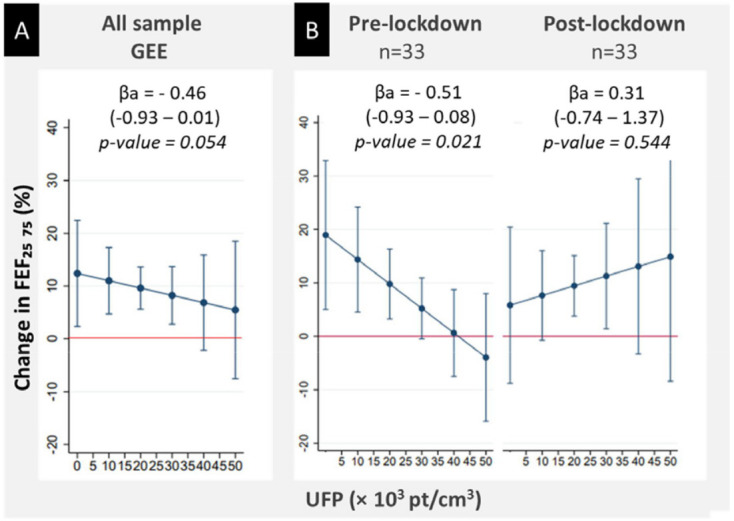
Associations between the relative change in FEF_25-75_ (forced expiratory flow at 25-75% of the forced vital capacity) and in-taxi ultrafine particles (UFP) concentration measured throughout the working day in the whole sample (A) and according to (B) the measurement period, in the PUF-TAXI project. ßa=adjusted coefficient; GEE=generalized estimating equations].The continuous outcome was the difference in percentage between FEF_25-75%_ measured at the end and the beginning of the first measurement day compared to the measurement at the beginning of the day). In A, the outcome was modeled using the GEE and in B using linear regression stratified by the measurement period (pre- versus pandemic period). ßa were calculated for 1000 particles/cm^3^ increase in average of UFP and 1 μg/m^3^ increase in average of BC, respectively. Model A was adjusted for age (years), having respiratory/allergic diseases (asthma or eczema or allergic rhinitis or a positive skin prick test) body mass index, the measurement period (before vs after the first lockdown), ambient temperature (°C), outdoor air quality (Atmo index), trip duration and temperature inside taxi vehicles (°C). Model B was adjusted for the same variables as A except for the stratification variable.

No association was observed between in-taxi particle concentrations and changes in lung parameters during the working day (table 2). However, the association of in-taxi UFP and the change in FEF_25–75%_ tended to be significant [β_adj_ 0.46 (95% CI-0.93‒0.01); P=0.054] and differed according to the measurement period (P for interaction <0.15). Indeed, during the pre-lockdown period a 1% decrease in FEF_25–75%_ pre- and post-working shift was significantly associated with each 10^3^ particles/cm^3^ increase in UFP inside taxi vehicles. No such association was found in the post-lockdown period (figure 3).

**Table 2:** Associations between the relative change in FVC, FEV_1_ and FEF_25-75_ and in-taxi UFP and BC concentrations measured throughout the working day, in the PUF-TAXI project. The continuous outcomes (difference in percentage between lung function parameters at the end and the beginning of the first measurement day compared to the measurement at the beginning of the day) were modeled using the generalized estimating equation. Adjusted beta coefficients (ß_adj_) were calculated for 1000 particles/cm^3^ increase in average of UFP and 1 μg/m^3^ increase in average of BC, respectively. [BC=black carbon; CI=confidence interval; FEV_1_= forced expiratory volume in one second; FEF_25-75_=forced expiratory flow at 25–75% of the FVC; FVC=forced vital capacity; UFP=ultrafine particles]

Dependent variable	Predictors	Generalized estimating equations	
		ß_adj_ (95% CI)	P for interaction ^a^
Δ FVC (%)	Model 1^b^		
	UFP (^×^103 pt/cm^3^)	0.07 (-0.08‒0.22)	0.787
	Model 2^b^		
	BC (μg/m^3^)	1.43 (0.003‒2.85)	0.744
Δ FEV_1_ (%)	Model 1^b^		
	UFP (^×^103 pt/cm^3^)	- 0.05 (-0.22‒0.11)	0.654
	Model 2^b^		
	BC (μg/m^3^)	0.23 (-1.34‒1.79)	0.612
Δ FEF_25-75_ (%)	Model 1^b^		
	UFP (^×^103 pt/cm^3^)	- 0.46 (-0.93‒0.01)	0.143
	Model 2^b^		
	BC (μg/m^3^)	-2.49 (-6.99‒2.02)	0.117

^a^ Interaction by the measurement period (pre- vs post-lockdown). ^b^ Adjusted for age (years), body mass index, having respiratory/allergic diseases (asthma or eczema or allergic rhinitis or a positive skin prick test), the measurement period (pre- vs post-lockdown), ambient temperature (°C), outdoor air quality (Atmo index), trip duration and temperature inside taxi vehicles (°C).

## Discussion

### Key results

This study adds new knowledge about the occupational exposure and health among taxi drivers, an understudied occupational group. The improvement in air quality inside vehicles, following the restrictions adopted during lockdown, changed the associations between in-vehicle pollutants and the respiratory health of taxi drivers. The incidence of nose irritation was positively associated with in-vehicle UFP and BC levels before lockdown, when pollutant levels were higher, whereas no significant association was found post-lockdown. Regarding lung function parameters, the decrease in FEF_25–75%_ during the working day was significantly associated with in-taxi UFP before, but not after lockdown. No such association was found with BC. By contrast, the incidence of eye irritation was significantly negatively associated with in-vehicle humidity, regardless of the pollutant concentrations and the measurement period.

### Variation in UFP and BC inside vehicles pre- and post-lockdown

As previously published (8), the mean concentrations of in-vehicle UFP and BC during working days decreased 1.7 times and 1.4 times post- compared to pre-lockdown, respectively. These results may be explained by the sudden decline in anthropogenic emissions during lockdown and remaining post-lockdown, in particular traffic flow (pre-lockdown: mean 778 (SD 44) versus post-lockdown: mean 703 (SD 90) vehicles per working hours; P<0.0001, Wilcoxon test for paired sample) (8). Indeed, since outdoor air pollutants enter vehicles, the decrease in particulate matter concentrations inside taxi vehicles can be partially attributable to the clear enhancement in ambient air quality as a result of restrictions implemented during COVID-19 lockdown (23–25). The reduction of in-vehicle UFP was also due to the variation of ventilation settings (8) as opening/closing windows and activating air recirculation are known determinants of in-vehicle UFP (12, 20, 26–28).

### Association between respiratory health and in-vehicle pollutants

Our findings show that the occurrence of nasal irritation in the overall sample during working days was significantly associated with in-vehicle UFP and BC concentrations while taking into account the repeated measurements. This result is consistent with our previous study, where an increase in the IQR of in-taxi UFP (20×10^3^ particles/cm^3^) and BC (1.75 µg/m^3^) was significantly associated with an increase in the incidence of nasal irritation [OR_adj_ 8.32 (95% CI 1.31‒52.6); OR_adj_ 7.97 (95% CI 1.11‒57.03)] during the working day, respectively (13). Interestingly, our results suggest that this relationship was modified by the measurement period (pre- and post-lockdown). This association remains significant for UFP, while BC tended to be significant before but not after the lockdown. One possible explanation is that the relatively low concentrations of in-vehicle UFP (17 versus 29×10^3^ particles/cm^3^; P<0.01) and BC (2.2 versus 3.2 µg/m^3^; P<0.001) post- compared to pre-lockdown were not sufficient to lead to nasal irritation. These results are in favor of dose–response relationships between in-vehicle short-term exposure to UFP and BC and nasal symptom. This supports a threshold hypothesis, where the threshold could be above 20×10^3^ particles/cm^3^ of UFP. Indeed, we should note when in-vehicle UFP were 13–21×10^3^ particles/cm^3^, no significant association was found with incident nasal irritation. However, the occurrence of nasal irritation in relation with in-vehicle UFP exposure was significant when UFP concentrations were 15–37×10^3^ particles/cm^3^. In addition, it has been found that when Australian participants commuted by bicycle using a route with low proximity to traffic, they were less exposed to UFP (19.1 versus 29.5×10^3^ particles/cm^3^; P≤0.001) and reported less nasal irritation mean 1.5 (SD 0.3) versus mean 1.9 (SD 0.2); P=0.007) compared to when they commuted using a route with higher proximity to traffic (29). Regarding BC, Guilbert et al (30) evaluated individual exposure to BC during four consecutive days and the respiratory health of 48 green space workers in Belgium. They found no significant association between BC levels and the reported respiratory symptoms, which is consistent with our results (30).

Moreover, the relationship between particle pollutants and nasal irritation could also be explained by their ability to deposit in the respiratory tract according to their physical properties (UFP <100 nm and BC component of PM_2.5_ with a diameter ≤2.5 µm). Experimental studies showed that fine particles (≤ 2.5 µm) and a large fraction of UFP (1–15 nm) are predicted to deposit in the extra thoracic airways, including nose. It is also reported that UFP appeared to be cleared less rapidly and completely from the respiratory tract than larger particles (31–33). This may explain why the association between nasal irritation and UFP concentrations is stronger than with BC.

Furthermore, UFP of 20–100 nm have the highest deposition efficiency in the alveolar region according to the International Commission on Radiological Protection (IRCP) models (31, 33). It is therefore not surprising that the increase in UFP concentrations inside vehicles was associated with a decrease in FEF_25–75%_ (only before lockdown, when pollutant levels were higher). No such associations were found with BC. These results are in line with our previous PUF-TAXI study (13) where the reduction in FEF_25–75%_ was associated with UFP but not with BC. By contrast, in this latter article, significant negative associations between in-vehicle UFP and FVC and FEV_1_ were also found. Several explanations can be proposed. First, with a small sample size of 33 taxi drivers, we suppose that the lack of statistical power made it difficult to detect the effect of in-vehicle UFP on FEV_1_ and FVC changes. Second, FEF_25–75%_ reflects the first change associated with airflow obstruction in small airways (34, 35). It should be noted that no taxi driver has reported changing in his smoking behavior between pre- and post-lockdown. The same pattern of associations between respiratory health and in-vehicle pollutants were observed when only comparing non- and ex-smokers.

Studies exploring the association between changes in lung function parameters and in-transit TRAP exposure, particularly to UFP and BC, remain few and their results are unclear (12, 36, 37). Future research is needed to confirm our results.

Furthermore, while there was no association between in-vehicle UFP/BC and eye irritation, as expected, the decrease in humidity inside taxi vehicles was significantly related to the occurrence of eye irritation. This result supports the findings of our previous study (13). Indeed, a recent review reported that the increase in indoor air humidity improves eye irritation symptoms (38).

### Strengths and limitations

This study supports the hypothesis of the short-term respiratory health impact of UFP. An important strength is the standardized method to assess exposure and health. Measurements were repeated by the same investigator, using the same robust devices, and following the same protocol, pre-and post-lockdown. The small sample size is a shortcoming of our study, however balanced by our study design including repeated measurements to increase the statistical power. It cannot be excluded that the high prevalence of atopy in these taxi drivers, could have influenced the results. Another limitation is that seasonal variation was not considered in the multivariable model, but we adjusted for the ambient temperature and in-taxi temperature.

### Concluding remarks

To our knowledge, this is the first study to investigate the effect of lockdown restrictions on in-vehicle air quality and respiratory health during the COVID-19 pandemic. Despite the relatively small sample size, the findings presented here support our previous results and show that the magnitude of the incidence of nasal irritation and decrease in lung function depends on the UFP concentrations the population is exposed to, suggesting a threshold hypothesis. This hypothesis needs further investigation.

### Ethical approval

PUF-TAXI project has been declared to the National Commission for Data Protection and Liberties (CNIL) with the following number: 2172757v0. The French Institutional Review Board approved it (Île-De-France III Ethics Committee 3593-RM; Research Ethics Number: 2018-A00811-54).

## Supplementary material

Supplementary material

## References

[1] Zhou P, Yang XL, Wang XG, Hu B, Zhang L, Zhang W et al. A pneumonia outbreak associated with a new coronavirus of probable bat origin. Nature 2020 Mar;579(7798):270–3. 10.1038/s41586-020-2012-7 32015507 PMC7095418

[2] Forster PM, Forster HI, Evans MJ, Gidden MJ, Jones CD, Keller CA et al. Erratum: Publisher Correction: Current and future global climate impacts resulting from COVID-19. Nat Clim Chang 2020;10(10):971. 10.1038/s41558-020-0904-z 32845944 PMC7427494

[3] Mousazadeh M, Paital B, Naghdali Z, Mortezania Z, Hashemi M, Karamati Niaragh E et al. Positive environmental effects of the coronavirus 2020 episode: a review. Environ Dev Sustain 2021;23(9):12738–60. 10.1007/s10668-021-01240-3 33558801 PMC7859094

[4] Marinello S, Butturi MA, Gamberini R. How changes in human activities during the lockdown impacted air quality parameters: A review. Environ Prog Sustain Energy 2021;40(4):e13672. 10.1002/ep.13672 34221243 PMC8237064

[5] Rodríguez-Urrego D, Rodríguez-Urrego L. Air quality during the COVID-19: PM_2.5_ analysis in the 50 most polluted capital cities in the world. Environ Pollut 2020 Nov;266(Pt 1):115042. 10.1016/j.envpol.2020.115042 32650158 PMC7333997

[6] Faridi S, Yousefian F, Janjani H, Niazi S, Azimi F, Naddafi K et al. The effect of COVID-19 pandemic on human mobility and ambient air quality around the world: A systematic review. Urban Clim 2021 Jul;38:100888. 10.1016/j.uclim.2021.100888 36536793 PMC9750834

[7] Arora S, Bhaukhandi KD, Mishra PK. Coronavirus lockdown helped the environment to bounce back. Sci Total Environ 2020 Nov;742:140573. 10.1016/j.scitotenv.2020.140573 32619844 PMC7323667

[8] Hachem M, Bensefa-Colas L, Momas I. Changes in air quality in-taxis and in working conditions of taxi drivers pre- and post-lockdown, during the COVID-19 pandemic in the Paris area. Indoor Air 2022 Jan;32(1):e12967. 10.1111/ina.12967 34866247

[9] Dominski FH, Lorenzetti Branco JH, Buonanno G, Stabile L, Gameiro da Silva M, Andrade A. Effects of air pollution on health: A mapping review of systematic reviews and meta-analyses. Environ Res 2021 Oct;201:111487. 10.1016/j.envres.2021.111487 34116013

[10] Orellano P, Reynoso J, Quaranta N, Bardach A, Ciapponi A. Short-term exposure to particulate matter (PM_10_ and PM_2.5_), nitrogen dioxide (NO_2_), and ozone (O_3_) and all-cause and cause-specific mortality: systematic review and meta-analysis. Environ Int 2020 Sep;142:105876. 10.1016/j.envint.2020.105876 32590284

[11] Li MH, Fan LC, Mao B, Yang JW, Choi AM, Cao WJ et al. Short-term Exposure to Ambient Fine Particulate Matter Increases Hospitalizations and Mortality in COPD: A Systematic Review and Meta-analysis. Chest 2016 Feb;149(2):447–58. 10.1378/chest.15-0513 26111257

[12] Hachem M, Saleh N, Paunescu AC, Momas I, Bensefa-Colas L. Exposure to traffic air pollutants in taxicabs and acute adverse respiratory effects: A systematic review. Sci Total Environ 2019 Nov;693:133439. 10.1016/j.scitotenv.2019.07.245 31374502

[13] Hachem M, Loizeau M, Saleh N, Momas I, Bensefa-Colas L. Short-term association of in-vehicle ultrafine particles and black carbon concentrations with respiratory health in Parisian taxi drivers. Environ Int 2021 Feb;147:106346. 10.1016/j.envint.2020.106346 33388565

[14] Graham BL, Steenbruggen I, Miller MR, Barjaktarevic IZ, Cooper BG, Hall GL et al. Standardization of Spirometry 2019 Update. An Official American Thoracic Society and European Respiratory Society Technical Statement. Am J Respir Crit Care Med 2019 Oct;200(8):e70–88. 10.1164/rccm.201908-1590ST 31613151 PMC6794117

[15] Lammers A, Janssen NA, Boere AJ, Berger M, Longo C, Vijverberg SJ et al. Effects of short-term exposures to ultrafine particles near an airport in healthy subjects. Environ Int 2020 Aug;141:105779. 10.1016/j.envint.2020.105779 32402984

[16] Paunescu AC, Casas M, Ferrero A, Pañella P, Bougas N, Beydon N et al. Associations of black carbon with lung function and airway inflammation in schoolchildren. Environ Int 2019 Oct;131:104984. 10.1016/j.envint.2019.104984 31301585

[17] Ohlwein S, Kappeler R, Kutlar Joss M, Künzli N, Hoffmann B. Health effects of ultrafine particles: a systematic literature review update of epidemiological evidence. Int J Public Health 2019 May;64(4):547–59. 10.1007/s00038-019-01202-7 30790006

[18] Samoli E, Rodopoulou S, Schneider A, Morawska L, Stafoggia M, Renzi M et al. Meta-analysis on short-term exposure to ambient ultrafine particles and respiratory morbidity. Eur Respir Rev 2020 Oct;29(158):200116. 10.1183/16000617.0116-2020 33115789 PMC9488642

[19] Yang Y, Ruan Z, Wang X, Yang Y, Mason TG, Lin H et al. Short-term and long-term exposures to fine particulate matter constituents and health: A systematic review and meta-analysis. Environ Pollut 2019 Apr;247:874–82. 10.1016/j.envpol.2018.12.060 30731313

[20] Hachem M, Saleh N, Bensefa-Colas L, Momas I. Determinants of ultrafine particles, black carbon, nitrogen dioxide, and carbon monoxide concentrations inside vehicles in the Paris area: PUF-TAXI study. Indoor Air 2021 May;31(3):848–59. 10.1111/ina.12779 33350528

[21] Miller MR, Hankinson J, Brusasco V, Burgos F, Casaburi R, Coates A et al.; ATS/ERS Task Force. Standardisation of spirometry. Eur Respir J 2005 Aug;26(2):319–38. 10.1183/09031936.05.00034805 16055882

[22] Textor J, Hardt J, Knüppel S. DAGitty: a graphical tool for analyzing causal diagrams. Epidemiology 2011 Sep;22(5):745. 10.1097/EDE.0b013e318225c2be 21811114

[23] AIRPARIF. COVID-19 et qualité de l’air AIRPARIF; 2021 Mai 2021. Contract No.: #03#04.

[24] Ikhlasse H, Benjamin D, Vincent C, Hicham M. Environmental impacts of pre/during and post-lockdown periods on prominent air pollutants in France. Environ Dev Sustain 2021;23(9):14140–61. 10.1007/s10668-021-01241-2 33519298 PMC7825385

[25] Magazzino C, Mele M, Schneider N. The relationship between air pollution and COVID-19-related deaths: an application to three French cities. Appl Energy 2020 Dec;279:115835. 10.1016/j.apenergy.2020.115835 32952266 PMC7486865

[26] Hachem M, Bensefa-Colas L, Lahoud N, Akel M, Momas I, Saleh N. Cross-sectional study of in-vehicle exposure to ultrafine particles and black carbon inside Lebanese taxicabs. Indoor Air 2020 Nov;30(6):1308–16. 10.1111/ina.12703 32496613

[27] Onat B, Şahin ÜA, Uzun B, Akın Ö, Özkaya F, Ayvaz C. Determinants of exposure to ultrafine particulate matter, black carbon, and PM2.5 in common travel modes in Istanbul. Atmos Environ 2019;206:258–70. 10.1016/j.atmosenv.2019.02.015

[28] Yu N, Shu S, Lin Y, Zhu Y. Assessing and reducing fine and ultrafine particles inside Los Angeles taxis. Atmos Environ 2018;181:155–63. 10.1016/j.atmosenv.2018.03.023

[29] Cole-Hunter T, Jayaratne R, Stewart I, Hadaway M, Morawska L, Solomon C. Utility of an alternative bicycle commute route of lower proximity to motorised traffic in decreasing exposure to ultra-fine particles, respiratory symptoms and airway inflammation--a structured exposure experiment. Environ Health 2013 Apr;12(1):29. 10.1186/1476-069X-12-29 23566176 PMC4177132

[30] Guilbert A, De Cremer K, Heene B, Demoury C, Aerts R, Declerck P et al. Personal exposure to traffic-related air pollutants and relationships with respiratory symptoms and oxidative stress: A pilot cross-sectional study among urban green space workers. Sci Total Environ 2019 Feb;649:620–8. 10.1016/j.scitotenv.2018.08.338 30176473

[31] Oberdörster G, Oberdörster E, Oberdörster J. Nanotoxicology: an emerging discipline evolving from studies of ultrafine particles. Environ Health Perspect 2005 Jul;113(7):823–39. 10.1289/ehp.7339 16002369 PMC1257642

[32] Jiang J, Zhao K. Airflow and nanoparticle deposition in rat nose under various breathing and sniffing conditions: a computational evaluation of the unsteady effect. J Aerosol Sci 2010 Nov;41(11):1030–43. 10.1016/j.jaerosci.2010.06.005 21076632 PMC2976565

[33] HEI. Understanding the Health Effects of Ambient Ultrafine Particles. Boston, MA: Health Effects Institute; 2013.

[34] Ciprandi G, Cirillo I, Klersy C, Marseglia GL, Vizzaccaro A, Pallestrini E et al. Role of FEF25-75 as an early marker of bronchial impairment in patients with seasonal allergic rhinitis. Am J Rhinol 2006;20(6):641–7. 10.2500/ajr.2006.20.2914 17181110

[35] Pellegrino R, Viegi G, Brusasco V, Crapo RO, Burgos F, Casaburi R et al. Interpretative strategies for lung function tests. Eur Respir J 2005 Nov;26(5):948–68. 10.1183/09031936.05.00035205 16264058

[36] Knibbs LD, Cole-Hunter T, Morawska L. A review of commuter exposure to ultrafine particles and its health effects. Atmos Environ 2011;45(16):2611–22. 10.1016/j.atmosenv.2011.02.065

[37] Knibbs LD, Morawska L. Traffic-related fine and ultrafine particle exposures of professional drivers and illness: an opportunity to better link exposure science and epidemiology to address an occupational hazard? Environ Int 2012 Nov;49:110–4. 10.1016/j.envint.2012.08.013 23010254

[38] Wolkoff P. Indoor air humidity, air quality, and health - An overview. Int J Hyg Environ Health 2018 Apr;221(3):376–90. 10.1016/j.ijheh.2018.01.015 29398406

