# Supplementary material for: The impact of COVID-19 lockdown restrictions on the short-term association between in-vehicle particulate pollutants and the respiratory health of Parisian taxi drivers
Source: Scand J Work Environ Health. 2023 Aug 29;49(6):367–74. doi: 10.5271/sjweh.4089 (PMC10782384; doi:10.5271/sjweh.4089)
Supplement: Supplementary material [file SJWEH-49-367-S001.pdf]

The impact of COVID-19 lockdown restrictions on the short-term association between in-vehicle particulate pollutants and the respiratory health of Parisian taxi drivers[1]

by Melissa Hachem, PhD, Lynda Bensefa-Colas, MD, Isabelle Momas, PhD [2]

1. SUPPLEMENTARY MATERIAL
2. Corresponding author: Prof. Isabelle MOMAS, Paris University, CRESS – INSERM, UMR_1153, INRAE, HERA team, 4 avenue de l'Observatoire, 75006 Paris, France. [E-mail: isabelle.momas@parisdescartes.fr]

Table of contents

TABLES AND FIGURES ....
EXPOSURE ASSESSMENT ....
QUALITY ASSURANCE AND DATA PROCESSING ....
REFERENCES ....

TABLES AND FIGURES

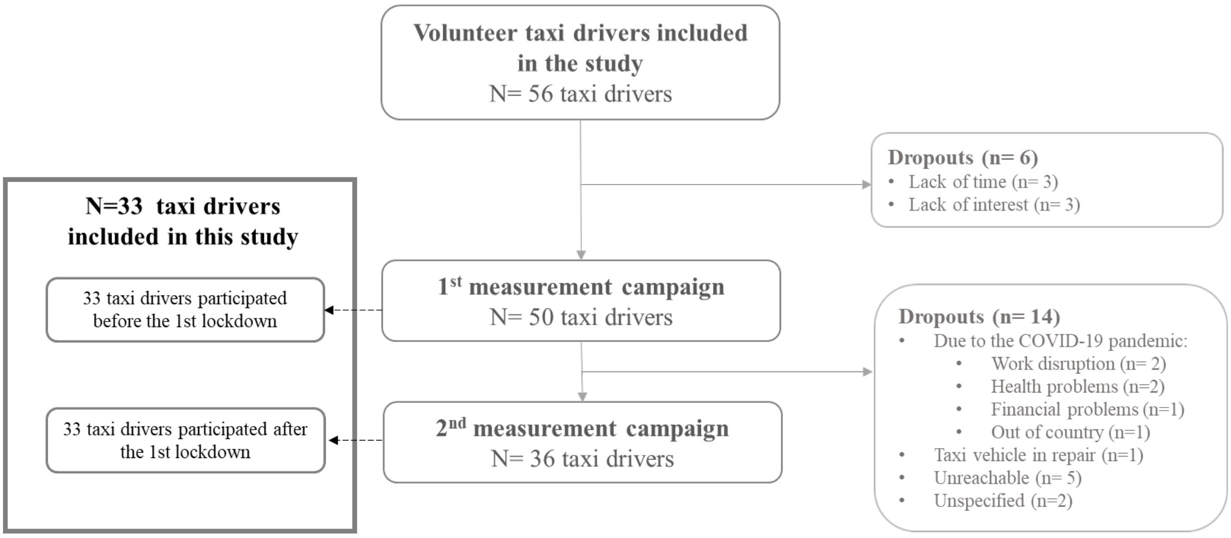

Figure S1. Study population flowchart of the PUF-TAXI study

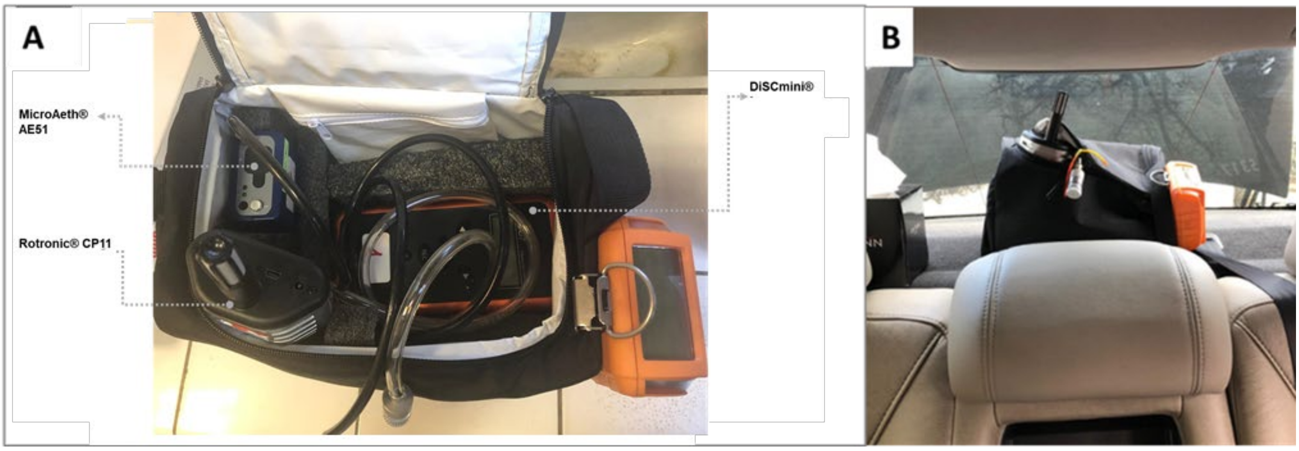

Figure S2. Photographs of the field measurement setup from PUF-TAXI study in Paris.

A: Carry case containing four measuring devices.
B: Carry case placed inside a taxi vehicle.

Table S1. General characteristics of the measurement campaigns, in the PUF-TAXI study

	Season	Start date	End date	Monitoring days	Temperature (°C)	Humidity (%)
**Pre-lockdown (2019)**	All seasons	14/02/2019	12/12/2019	33	11.9 ± 4.9 [3.3; 22.9]	73 ± 10 [53; 95]
	Cold season (Winter, autumn)	14/02/2019	12/12/2019	21	10.2 ± 4.5 [3.3; 19.1]	77 ± 9 [63; 95]
	Warm season (Spring)	22/03/2019	07/06/2019	12	14.9 ± 4.1 [7.5; 22.9]	66 ± 9 [53; 82]
**Post-lockdown (2020)**	All seasons	02/07/2020	09/12/2020	33	18.6 ± 7.3 [1.5; 33.1]	61 ± 19 [30; 93]
	Cold season (Autumn)	28/09/2020	09/12/2020	12	10.8 ± 5.1 [1.5; 17.6]	82 ± 9 [67; 93]
	Warm season (Summer)	02/07/2020	22/09/2020	21	23 ± 3.8 [17.2; 33.1]	49 ± 12 [30; 68]

Table S2. Baseline characteristics of Parisian taxi drivers, in the PUF-TAXI study (N= 33).

	**Mean ± SD**	**n/N (%)**
**General characteristics**		
Age (years)	51.6 ± 7.2	-
Smoking status		
Smoker [a]		5/33 (15)
Ex-smoker [b]		14/33 (42.5)
Never smoker		14/33 (42.5)
**Job-related characteristics**		
Job tenure (years)	15.5 ± 7.4	-
Working hours per day	8.8 ± 2.1	-
Working days per week	5.3 ± 1.4	-
**Health data at the medical inclusion visit**		
BMI (kg/m^2)	26.1 ± 3.9	-
Skin prick tests [c]		
Atopy by a positive SPT	-	13/24 (54)
**Reported respiratory and allergic diseases**		
Asthma ever [d]	-	4/33 (12)
Eczema ever	-	6/32 (19)
Allergic rhinitis ever [e]	-	10/32 (31)
Atopy ever	-	10/33 (30)
**Other reported diseases**		
Hypertension	-	5/33 (15)
Diabetes	-	1/33 (3)
GERD	-	24/33 (73)
**Reported irritations in the past year**		
Nose irritations [f]	-	13/32 (41)
During work	-	*13 (100)*
During work and reduced during vacation	-	*4 (31)*
Eye irritations [g]		
During work	-	*13 (100)*
During work and reduced during vacation	-	*6 (46)*
Throat irritations [h]		
During work	-	*10 (91)*
During work and reduced during vacation	-	*4 (36)*

BMI: body mass index; **GERD**: gastroesophageal reflux disease; **SD**: standard deviation; **SPT**: skin prick test.

a. **Smoker**: current smoker or had quit smoking for less than twelve months
b. **Ex-smoker**: have quit smoking for at least twelve months
c. **SPT** is considered positive if the wheal diameter ≥ 3 mm and at least half the size of the positive histamine control (1)
d. **Asthma ever** is defined as having a childhood asthma (diagnosis between 5 – 14 years 'old)
e. **Allergic rhinitis** is defined as having at least one of the following symptoms: congestion, rhinorrhea, sneezing, nasal itching, and nasal obstruction without any infectious triggers and having a hay fever or an allergy to pollen or other allergens (2)
f. **Nose irritations**: sneezing, stuffy or runny nose, itchy nose without any infectious triggers
g. **Eye irritations**: redness, watery or itchy eyes without any infectious triggers
h. **Throat irritations**: hoarseness, irritation, and itching without any infectious triggers
i. **Chronic cough is** defined clinically as having cough every day for at least 3 consecutive months (3)
j. **Chronic phlegm** is defined clinically as having phlegm every day for at least 3 consecutive months (3)

None of the participants have chronic bronchitis, emphysema, chronic obstructive bronchopneumopathy

Table S3. Comparison of the measurement campaign characteristics pre- and post-lockdown in the Paris area, in the PUF-TAXI study

		**Pre-lockdown (n=33)**		**Post-lockdown (n=33)**		***p*-value**
		Mean ± SD	N (%)	Mean ± SD	N (%)	
**Background factors**	Ambient temperature (°C)	12 ± 5		19 ± 7		0.001[a]
	Ambient relative humidity (%)	73 ± 10		61 ± 19		0.008 [a]
	Outdoor air quality (Atmo index) [b]					
	Very good or good [1,2,3,4]		26 (79)		25 (76)	0.796 [c]
	Moderate or poor [5,6,7]		7 (21)		8 (24)	
	Traffic flow [d]	778 ± 44		703 ± 90		<0.0001 [e]
**Working conditions**	In-taxi temperature inside vehicle (°C)	22 ± 4		25 ± 5		0.009 [a]
	In-taxi relative humidity (%)	44 ± 11		41 ± 13		0.247 [a]
	In-taxi CO_2 (ppm)	1160 ± 661		1239 ± 968		0.422 [e]
	Trips duration (hr.)	5 ± 2		4 ± 2		0.001 [a]
	Distance travelled (km)	144 ± 51		126 ± 141		0.004 [e]
	Time of OP relative to the trips duration (%)	38 ± 33		53 ± 37		0.039 [a]
	Time of AC activation relative to the trips duration (%)	48 ± 40		41 ± 42		0.364 [a]
	Time of AR activation relative to the trips duration (%)	24 ± 41		11 ± 27		0.230 [e]

AC: air-conditioning; **AR**: air recirculation; **OP**: opening windows; **SD**: standard deviation

a. Paired sample T-test
b. Atmo index is based on four air pollutant concentrations: SO_2, NO_2, O_3, and PM_{10}. For each of these pollutants, a sub-index is calculated and the Atmo index of the day is equal to the highest of the four sub-indices, scaled from 1 (very good) to 10 (very bad) (https://www.airparif.asso.fr)
c. McNemar's test for paired sample
d. Traffic flow is the hourly mean number of vehicles averaged over 24 hours, during each sampling day
e. Wilcoxon test for paired sample

Table S4. Comparison of the incidence of the reported symptoms in Parisian taxi drivers pre- and post- lockdown, in the PUF TAXI study (N=33).

**Reported symptoms during the working day**	**Pre- lockdown n (%)**	**Post- lockdown n (%)**
**Nasal irritations [a]**	6 (18)	4 (12)
**Eye irritations [b]**	5 (15)	2 (6)
**Eye or nose irritation**	11 (33)	6 (18)

Note:

An incident irritation was defined as the nasal or eye irritation occurring during the working day or getting worse compared to the start of the day (the symptom intensity scale during the working day > the symptom intensity scale at the start of the working day).

a. Nasal problems: sneezing, stuffy or runny nose, itchy nose.

b. Eye problems: redness, watery or itchy eyes.

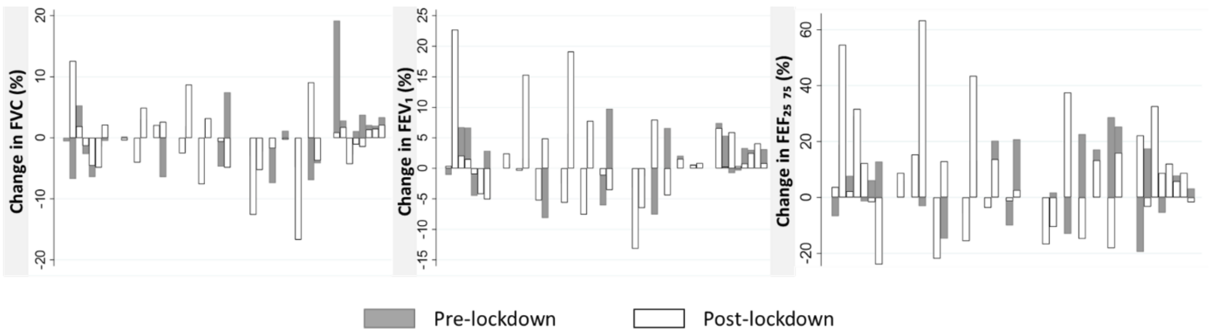

Figure S5. Variation of lung function parameters in Parisian taxi drivers between pre- and post-lockdown, in the PUF-TAXI study (N=33)
$FEF_{25-75\%}$: forced expiratory flow at 25-75% of the forced vital capacity; **FEV_1**: forced expiratory volume at 1 second; **FVC**: forced vital capacity
Each bar represents a participant.

EXPOSURE ASSESSMENT

During each sampling day, the taxi driver carried a carry case containing the measuring instruments (Figure 1). Ultrafine particles (UFP) and black carbon (BC) were measured by the Diffusion Size Classifier Miniature (DiSCmini®- DM, Wohlen, Switzerland and commercialized by Testo SE &Co. KGaA, Titisee-Neustadt, Germany) and the microAeth® Model AE51 (MA, Aethlabs, San Francisco, California, USA), respectively. Since the aerosol sampling is distant from the DiSCmini® and the microAeth® original inlets, the sampling was made by tubes, supplied by the manufacturer, placed outside the case, and connected to the devices (Figure 1S). CP11® (Michel Instruments, Lyon, France) was used to monitor temperature, humidity, and carbon dioxide (CO_2) inside the vehicle. All the devices were logged at one min intervals. We should note that the same set of devices was given to all the taxi drivers.

QUALITY ASSURANCE AND DATA PROCESSING

Exposure measurement: Before the beginning of each survey day, the proper functioning of all the devices was checked according to the manufacturers. They were turned on for several minutes to stabilize before placing them in the vehicle. After each sampling day, DM and MA tubes were rinsed with filtered water and dried by pure air using a compressor. MA quartz filter was changed, and DM impactor was cleaned for one hour in an ultrasonic bath (Branson Model 1800, Fisher Scientific Co.L.L.C., Pittsburgh, PA, USA) with pure water to maintain measurement integrity. DM and MA were sent to their manufacturers on their calibration due dates.

Regarding data processing, records were downloaded from their respective equipment *via* their softwares (listed above) and were closely checked after being converted to Microsoft Excel files. Negatives BC values caused by low BC concentrations were corrected by the Optimized

Noise-reduction Averaging method available on the supplier's website (www.aethlabs.com). For all the other readings, suspicious data were removed.

Missing data: About 13% of the UFP data (concentration recorded per min) were lost due: (1) to the limited battery life of the DM (8 hours), (2) the sudden UFP's level change and (3) the high temperature inside the taxis during the hot season. Regarding MA and CP11, the percentages of the missing data were 3% and 2%, respectively.

Health assessment: a portable spirometer (Spirobank II®, MIR, Italy) was used according to the American Thoracic Society and the European Respiratory Society (ATS/ERS) guidelines (21). After explaining the appropriate technique of the test procedure, each driver performed in a sitting position wearing a nose clip, at least three satisfactory maneuvers. Following these guidelines, the lung function parameters of the best expiratory flow-volume curve were chosen.

All data were entered on a digital platform - electronic case report form (CleanWeb™; e-CRF) ensuring data management, quality, and security.

REFERENCES

1. Helaskoski E, Suojalehto H, Kuuliala O, Aalto-Korte K. Occupational contact urticaria and protein contact dermatitis: causes and concomitant airway diseases. Contact Dermat 2017 Dec;77(6):390–6. https://doi.org/10.1111/cod.12856.
2. Keil U, Weiland SK, Duhme H, Chambless L. The International Study of Asthma and Allergies in Childhood (ISAAC): objectives and methods; results from German ISAAC centres concerning traffic density and wheezing and allergic rhinitis. Toxicol Lett 1996 Aug;86(2-3):99–103. https://doi.org/10.1016/0378-4274(96)03680-6.
3. Mirza S, Clay RD, Koslow MA, Scanlon PD, editors. COPD guidelines: a review of the 2018 GOLD report. Mayo Clinic Proceedings; 2018: Elsevier.
